# Exploring the Relationship Between Hospital Service Quality, Patient Trust, and Loyalty From a Service Encounter Perspective in Elderly With Chronic Diseases

**DOI:** 10.3389/fpubh.2022.876266

**Published:** 2022-05-25

**Authors:** An-Jin Shie, Yung-Fu Huang, Guang-Yu Li, Wen-Yi Lyu, Ming Yang, You-Yu Dai, Zhao-Hui Su, Yenchun Jim Wu

**Affiliations:** ^1^College of Business Administration, Huaqiao University, Quanzhou, China; ^2^School of Economics and Management, Huaiyin Normal University, Huai'an, China; ^3^International College, Krirk University, Bangkok, Thailand; ^4^Department of Marketing and Logistics Management, Chaoyang University of Technology, Taichung, Taiwan; ^5^Shandong Holyscape Marketing Research & Consulting Co., Ltd, Jinan, China; ^6^International Business School, Shandong Jiaotong University, Jinan, China; ^7^Graduate Institute of Global Business and Strategy, National Taiwan Normal University, Taipei, Taiwan; ^8^College of Humanities and Arts, National Taipei University of Education, Taipei, Taiwan

**Keywords:** hospital service quality, trust, service encounter, doctor-patient loyalty, SERVQUAL

## Abstract

Based on the service encounter perspective, this study combines theoretical foundations for such factors as service quality and the characteristics of the hospital service industry to develop a research model scale to investigate whether the quality of hospital services affects patients' perceptions of health service encounters, trust, and loyalty. Nowadays, with the advancement of medical technology, patients pay more attention to the quality of medical services and good service encounters provided by healthcare professionals in order to establish positive patient relationships; hospitals need to improve their own service quality and establish good patient trust relationships so that doctor-patient satisfaction and loyalty can be improved. In a review of related literature, this study found that most past studies focused on issues related of quality of medical services and patient satisfaction, but ignored those related to the relationship between medical service encounters and patient trust and loyalty, as well as the lack of scientific measurement markers for service encounters in the Chinese medical service industry. Therefore, this study uses the Service Encounter Perspective and Service Quality Theory Development Research Scale to collect and analyze data for a typical case of a Chinese tertiary hospital. Finally, this study explores the relationship between the four variables of service quality, service encounter, trust, and loyalty by means of a questionnaire and statistical analysis of the data. Finally, it is concluded that the higher the service quality of the hospital, the higher the customer trust, the higher the service encounter, and in the greater the doctor-patient loyalty.

## Introduction

The original purpose of the medical service industry is to meet the medical needs of the society and to solve the problems of old age and sickness. Currently, medical service providers have adopted a patient-centric business strategy, and it has become a challenge and an important evaluation marker for medical service providers to improve their service encounter and quality to meet the needs of patients and to increase their trust in hospitals (1–3). The report of the 19th National Congress of the Chinese Communist Party clearly states that the quality of medical care should be continuously improved to enhance the health of the people (4). In addition, the Chinese people's concern for the quality and health of hospital services has made the service quality and attitude of medical staff a relevant topic throughout all of society (5). However, due to the influence of the old medical system, China's medical and health system is undergoing profound changes, giving rise to many problems, which has resulted in a gap in the people's demand and expectation for medical services. This is an important factor causing social disharmony and instability.

Therefore, public hospitals are focusing their efforts on medical services, which has practical and theoretical implications for the continuous improvement of service quality and sustainable and healthy development of public hospitals. To reduce the occurrence of medical disputes, public hospitals must improve the quality of services with a focus on “patient safety.” The improvement of patients' expectations of the quality of medical services will build long-term trust between doctors and patients and lay the foundation for a good branding of the hospital (6, 7). At the same time, patients' evaluation of hospital services can be used to assess the overall strength of the hospital in terms of service excellence, staff technical standards, and management levels (8). In view of customers' demand for hospital service quality and the total national health insurance budget limit, public hospitals must undergo the following changes to be sustainable: (**i**) improve the quality of services (6, 7); (**ii**) pay attention to patient satisfaction after medical service encounter (1, 9); and (**iii**) improve patient and family satisfaction and loyalty to medical care (10). Among these, medical service encounter is the interaction between frontline healthcare workers and patients, and is also the most important part of service quality to patients. When strengthening competitiveness in hospitals, doctors and nursing staff are the frontline medical staff that face and have direct contact with patients. Therefore, medical service encounter is a key factor in maintaining and strengthening the doctor-patient relationship, thereby creating patient loyalty, and thus is a key factor in the survival of today's hospitals.

Medical service encounters happen when patients need highly professional medical services. In particular, the elderly or chronic disease patients require long-term medical services, including dialysis, Alzheimer's disease prevention, diabetes prevention, and hypertension prevention (6). These medical services, involving several encounters (e.g., physician, caregiver, service personnel, hospital space and equipment service encounters), are periodicity used by patient (11). The medical service encounter could stimulate patients' positive experience of medical services and facilitate more interaction and two-way communication between patients and medical staff.

A review of the literature on service quality in the healthcare industry has primarily focused on (**i**) healthcare service quality and patient loyalty (12, 13); (**ii**) healthcare service quality and patient satisfaction (11, 14, 15); (**iii**) patient satisfaction and patient loyalty (12, 13, 16, 17); (**iv)** the physician-patient relationship and patient loyalty (18–20); and (**v**) the quality of the patient-patient relationship and patient loyalty (13, 16). In previous studies, research gaps still exist regarding whether the “service encounter” in healthcare can improve the relationship between patient quality, trust, and loyalty to hospital services, and aid in the development of the quality assessment by hospital service encounter. Therefore, from the viewpoint of healthcare encounters, the “Service Encounter Assessment Model” proposed by Chang et al. (21) and Gonzalez (2) was applied in this study to develop a research model and assessment scale to explore the relationship between the quality of hospital service and patients' trust and loyalty to hospital services after receiving medical service encounters. The objectives of this study, which was conducted in a large general public hospital, are as follows.

(1) To develop a service encounter and service quality scale that is suitable for the healthcare industry, using the “service encounter” perspective in conjunction with hospital service characteristics and service quality.
(2) To examine whether healthcare encounters enhance patient trust and loyalty.
(3) To explore whether improvements in hospital service quality positively affect patient contact, trust and loyalty to healthcare services.

## Literature Review

### Service Encounters

The service encounter is the most direct marker for patients and families to assess the quality of healthcare services, and is an important interface for patients to experience the professional competence of healthcare services. Stock et al. (22) proposed the theory of transformational behavior and applied it to the service industry, in which service encounters mainly involve human contact as the interface. They explored the different personal experiences of customers in interpersonal, technological, and professional service encounters. If a consumer has an unpleasant service encounter experience during the interaction with the service provider, “switching behavior” will occur (1, 23). Consumers seek out suitable and trustworthy service providers in a proactive manner. Therefore, the service encounter can be an important interface to enhance customer trust. Kim et al. (3) stated that medical services are human-centric, high contact, highly customizable, highly professional and onsite services that need to be patient-driven to enhance patient satisfaction, trust, and loyalty. Gonzalez (2) noted that patient-physician interactions include all healthcare encounters, as well as encounters with healthcare-staff-related equipment. The interactions between doctors, caregivers, and patients, as well as the spaces and equipment that patients are exposed to, are considered integral to the quality of healthcare services and affect patient satisfaction, trust, and loyalty. In conclusion, this study proposes that providing friendly healthcare encounters in hospitals can enhance patient trust, maintain a good doctor-patient relationship, and further increase patient loyalty.

### Quality of Hospital Services

The service quality model “SERVQUAL” was developed by Parasuraman et al. (24) to explain the degree of difference between the perceived service quality (PSQ) and customer expectations in the service delivery process (10, 25). Parasuraman et al. (24) proposed SERVQUAL, which comprises 10 measurement dimensions: tangibles, reliability, responsiveness, credibility, courtesy, security, competence, communication, access, and understanding. Later, they employed factor analysis to reduce the 10 dimensions of the previous service quality model to 5 measurement dimensions: tangibles, reliability, responsiveness, assurance, and empathy, making it more suitable for application in other service industries (7, 10). Hsu (9) and Anabila et al. (26) proposed that the five dimensions of healthcare quality have positive effects on the customer's experience after encountering the service, and can also affect customer confidence and loyalty. Poor service quality can also lead to a loss of customer confidence and loyalty in addition to causing a poor post-encounter experience (27), and ultimately result in customer consumption switching behavior. Therefore, this study concludes that good service quality in hospitals will not only enhance patients' service encounter experience, but also improve patients' trust and loyalty to hospitals.

### Patient Trust

Patients' trust stems from the real feelings that patients experience from the honesty, integrity and reliability of doctors and caregivers after encountering healthcare services, and patients tend to have higher trust and loyalty if they are more satisfied with their healthcare encounters. Sbaffi et al. (28) and Adeleke et al. (29) pointed out that trust refers to the patient's belief that his or her health needs can be adequately met by the healthcare provider, and the intention to build confidence and goodwill and willingness to establish a long-term relationship. The level of customer trust shows a linear relationship to customer loyalty, with a psychological state of trust and dependence arising when customers' actual service perceptions exceed expectations. Fatonah (18) mentioned that in the field of medical services, patients generate “quality perception/price perception > satisfaction > trust > loyalty,” an in particular, when patients are regularly exposed to medical services, their “trust” has the strongest impact on “loyalty.” Druica et al. (30) and Castaldo et al. (31) conducted interviews with patients regarding healthcare service encounters, and the nine services that are most important to patients' confidence were compiled and further developed into markers. The study also pointed out that patient confidence changes due to the professionalism of doctors and caregivers during healthcare service encounters. At the same time, the quality of service provided by doctors and caregivers can also cause changes in patient confidence. In this study, the nine patient trust markers of Druica et al. (30) and Castaldo et al. (31) were used as a reference for the patient trust scale. The markers in this study were further modified according to the characteristics of medical services. In summary, patients have higher loyalty when they have higher trust in hospital services after being exposed to medical services; contrariwise, patients have lower loyalty when they have lower trust in hospital services.

### Patient Loyalty

Yang and Yuan (32) argue that customer loyalty will affect customers' buying behavior and that customer satisfaction is just an attitude expression and may not change buying behavior. Companies are familiar with the fact that as customer acquisition, customer retention, and customer profit growth are areas that companies need to work on, building customer loyalty to maintain a competitive advantage in the market is an important issue. Customer loyalty is one of the best intangible assets an organization can have, both at the attitudinal and behavioral levels, and it is a huge potential differentiator that can be a source of increased competitive advantage (33). However, for a company to have a high competitive advantage, it is important to carefully analyze and communicate effectively with each customer in order to fulfill the commitment to them, which is very important in the ever-changing market and helps to increase the level of customer satisfaction and loyalty (34). Huang et al. (35) noted that patient loyalty assessment can be divided into two aspects: attitudinal loyalty and behavioral loyalty. In terms of attitudinal loyalty, the main measures are: (1) primary willingness to visit: the tendency of patients to prefer a particular hospital when they have a medical need; (2) revisit willingness: the willingness of patients to visit the hospital again; and (3) loyalty-derived behavior: the willingness to recommend the hospital to others. With regards to behavioral loyalty, the main markers are the frequency of patients' visits to that hospital and the total number of visits.

## Hypothesis Development

### Hospital Service Quality, Service Encounter, Trust and Loyalty

Kim et al. (3) conducted an empirical study focusing on assessing the medical services' quality provided at a complementary and alternative medicine-oriented hospital using the service encounter approach, and analyzed the influence of treatment effectiveness on patient loyalty. The results indicated that the physician in a service encounter and service quality had a positive effect on treatment effectiveness. Yu et al.'s (27) study indicated that the impression of the facilities and environment in a service encounter directly impacted patient's satisfaction rates for interpersonal-based medical service encounters; in contrast, treatment effectiveness positively affected satisfaction regarding the medical service quality. Gonzalez's (2) study indicated that the most patient-physician interactions generated by healthcare encounters and exposed by patients were (1) healthcare-staff-related equipment; (2) interactions between doctors, caregivers, and patients; and (3) spaces and equipment. The service encounters in healthcare should be considered integral to the healthcare services quality and to their effect on patient satisfaction, trust, and loyalty. From the above statements, the following hypotheses were developed:

H1: **Hospital service quality** has a significant positive effect on **service encounter**.
H2: **Hospital service quality** has a significant positive effect on **trust**.
H3: **Hospital service quality** has a significant positive effect on **doctor-patient loyalty**.

### Service Encounter, Trust and Loyalty

Previous studies (2, 3) on service encounters from the perspective of patient-centered needs and concluded that interactions between doctors and caregivers, spaces, equipment that patients used, and service personnel quality influence patients' trust and loyalty to service encounters. The studies of Druica et al. (30) and Castaldo et al. (31) emphasize that healthcare service encounters are crucial to patients' confidence improvement. These studies also found that patient confidence differs depending on the professionalism of doctors and caregivers during healthcare service encounters. Simultaneously, the service encounter provided by doctors and caregivers can also cause increasing patient loyalty. Taken together, the following hypotheses were developed:

H4: **Service encounter** has a significant positive effect on **doctor-patient loyalty**.
H5: **Service encounter** has a significant positive effect on **doctor-patient trust**.

### Patient Trust and Loyalty

Regarding the relationship between patients' trust and loyalty (13, 16–18, 36), findings show that the degree of patients' trust positively influences loyalty, and (13, 16, 17) prior trust directly and positively affects consequent satisfaction. Alhatti (16) and Fatima et al. (13) found that patients' trust under the perfect hospital service quality or service encounter can stimulate loyalty positively. Sbaffi et al. (28) and Adeleke et al. (29) showed that patients' trust results from the doctors and caregivers that provide adequate service to meet patients' health needs. Doctors and caregivers' goodwill and willingness can establish a long-term relationship of loyalty and trust. The level of patients' trust shows a linear relationship to their loyalty, with a psychological state of trust and dependence arising when patients' actual received service quality or service encounters exceed expectations. Medical service is an intangible product of service encounters; both medical care personnel and general service personnel must develop a trusting relationship with patients to enhance patient loyalty. Based on the above statements, the following hypothesis was developed:

H6: **Doctor-patient trust** has a significant positive effect on **doctor-patient loyalty**.

## Methodology

### Hypotheses Presented in a Model Diagram

**Figure 1:**
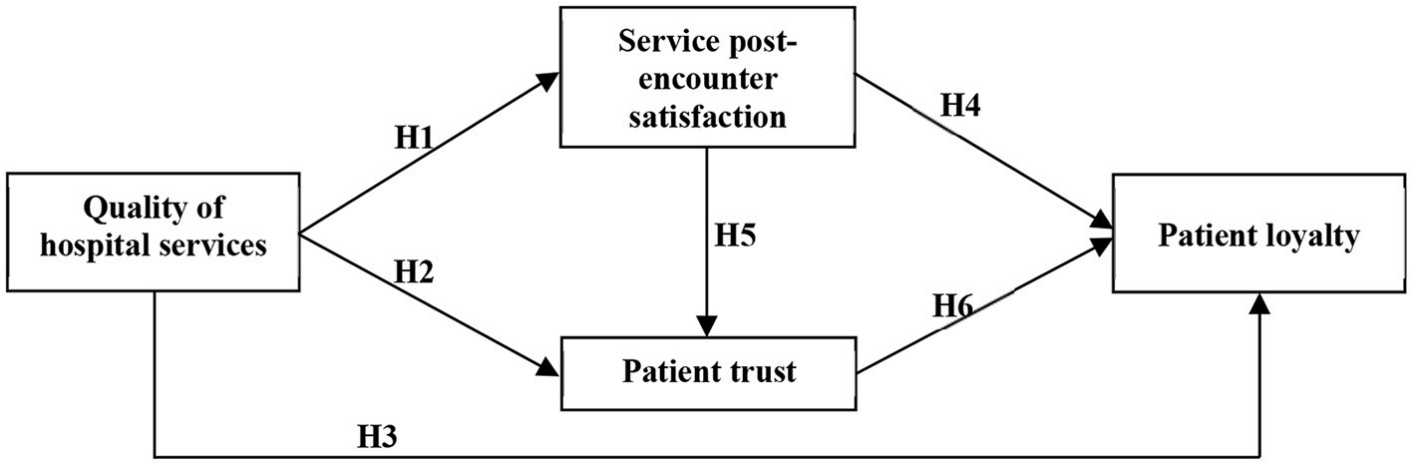
Research model.

In order to achieve the study objectives, this study focuses on the “service encounter” perspective and further develops the hospital service encounter and quality measurement scale by combining the characteristics of hospital services to investigate whether the quality of hospital services has a positive impact on patients' perceptions of healthcare services, trust and loyalty. Therefore, this study proposes a study model (Figure 1) consisting of hospital service quality, service encounter, and patient trust and loyalty dimensions. With regards to hospital service quality, this study is mainly based on the “service quality model” proposed by Parasuraman et al. (24), which is combined with the five measurement dimensions of medical service quality proposed by Chang et al. (37). With regards to service encounter, this study utilizes the “Service Encounter Assessment Model” Chang et al. (21) and Gonzalez (2) to develop a study model and scale, and modified it to take into account the characteristics of the healthcare service industry. This study also references the nine patient trust measurement markers Gabay (36) and Castaldo et al. (31) and the three trust measurement markers Huang et al. (35). This study also integrates the trust and loyalty-related components valued by patients in the healthcare service industry with the markers developed by the aforementioned scholars. Finally, this study utilizes a five-point Likert scale, where the scores 1–5 indicate *strongly disagree, disagree, no opinion, agree*, and *strongly agree*, respectively, as shown in Table 1.

**Table 1:** Hospital Service Encounter and Quality Measurement Scale.

**Dimension**	**Measurement items**	**References**
Tangibles	1. The hospital has safe facilities.	(11, 14, 15, 26, 37–39)
	2. The hospital has a barrier-free space design.	
	3. The hospital has a neat and comfortable environment.	
	4. The hospital has modern medical facilities.	
	5. The medical staff of the hospital are professionally dressed.	
Reliability	6. The doctors and nurses at this hospital can provide skilled and professional services.	
	7. Doctors at this hospital can give detailed information about the patient's condition and treatment.	
	8. The hospital's medical and nursing staff can provide prompt services to patients.	
	9. The medical staff at this hospital have a serious and genuine attitude toward their work service.	
Responsiveness	10. The hospital's medical staff is able to deal with patients' problems in a timely and prompt manner.	
	11. The hospital is able to clearly inform patients of the consultation and treatment process.	
	12. The hospital's doctors and nurses do not let their busy schedules delay meeting the patient's needs.	
	13. The medical staff of this hospital can provide medical advice in a timely and appropriate manner.	
	14. The medical staff at this hospital are always very helpful to patients.	
Assurance	15. The hospital's medical staff makes patients feel safe about their visits.	
	16. The professional performance of the medical staff at this hospital makes patients feel confident.	
	17. The doctors and nurses at this hospital have sufficient expertise to answer patients' questions.	
	18. The medical and nursing staff at this hospital generally have a good service attitude and courtesy.	
Empathy	19. The medical staff of this hospital will prioritize the best interests of the patient.	
	20. The medical staff at this hospital is sensitive to the privacy of the patient's visit.	
	21. The hospital's medical clinic hours are convenient for the average patient.	
	22. The hospital's medical staff is able to meet the special medical needs of patients.	
Physician **service encounters**	1 I think the doctors at that hospital will address my concerns.	(2, 21)
	2 I think the doctors at that hospital are reliable for me.	
	3 I think the doctors at this hospital have a good consultation attitude.	
	4 I think that the doctors at this hospital possess medical expertise.	
	5 I think the doctor at that hospital would recommend appropriate medication for me.	
	6 I believe that the doctors in that hospital show empathy for their patients.	
	7 I think the doctors at that hospital would tell the patients the treatment plan.	
Caregiver **service encounters**	8 I feel that the nurses at that hospital are reliable for me.	
	9 I think the nurses at that hospital will relieve me of my worries.	
	10 I believe that the nurses at this hospital show empathy for their patients.	
	11 I think the nurses in this hospital have a good attitude toward patient service.	
	12 I think the nurses in this hospital are professional.	
Service personnel **service encounters**	13 I find that the service staff at the hospital are reliable for me.	
	14 I think the service staff at that hospital can relieve me of my worries.	
	15 I believe that the attitude of the service staff at this hospital is beneficial to patients.	
	16 I feel that the service process in this hospital is efficient and convenient for patients.	
Space and equipment **service encounters**	17 I believe that the medical office of the hospital will give consideration to the privacy of the patients.	
	18 I think there are clear markers in the interior of that hospital.	
	19 I find that the toilets in the hospital to be clean and hygienic.	
	20 I find that the consultation rooms in the hospital to be spacious and bright.	
	21 I believe that the hospital has advanced medical equipment.	
Patient trust	1 I have complete faith that the doctors here will provide the best course of treatment for me.	(31, 36, 40)
	2 The doctor who sees me at this hospital will do his or her best to provide the medical care I need.	
	3 I am not worried about putting my treatment solely in the hands of the doctors here.	
	4 The doctor who treated me at this hospital was very careful and attentive.	
	5 The doctors at that hospital are honest about all treatment options available to me.	
	6 The doctors at that hospital will think of the best way to treat me.	
	7 I believe the doctors at that hospital would never mislead me about anything.	
	8 I trust that the doctors at this hospital will usually use their best medical skills and efforts to treat their patients.	
	9 Overall, I can trust the doctors here completely.	
Patient loyalty	1 I prefer to go to the hospital for medical services	(35)
	2 I will choose the medical services provided by this hospital in the future.	
	3 I am happy to recommend the medical services provided by the hospital to friends and family.	

The above discussion and literature suggest that the quality of healthcare services affects patients' perceptions of their satisfaction with hospital service encounters, as well as their trust and loyalty toward doctors and healthcare workers. In addition, the interaction between doctors, nursing staff, and service providers with patients, as well as the space and equipment to which the patients are exposed, are healthcare encounters that enhance patient loyalty and trust. This study proposes the following hypotheses based on the above literature and research model.

H1: Hospital service quality has a significant positive effect on service encounter.
H2: Hospital service quality has a significant positive effect on trust.
H3: Hospital service quality has a significant positive effect on doctor-patient loyalty.
H4: Service encounter has a significant positive effect on doctor-patient loyalty.
H5: Service encounter has a significant positive effect on doctor-patient trust.
H6: Doctor-patient trust has a significant positive effect on doctor-patient loyalty.

### Study Sample and Data Collection

According to the data released by the Statistical Information Center of the National Health and Health Commission, there are 1,441 grade-A tertiary hospitals in China. Among the proportion of Geriatrics (including elderly care services) set up in the grade-A tertiary hospitals in Jiangsu Province, 65.4% accounts for about 39% of the national total, ranking first in the country. Meanwhile, 77.4% of those over 60 years old and 85.3% of those over 80 years old have chronic diseases that require long-term medical service encounters.

Therefore, this study utilized the 2021 list of hospitals in Jiangsu Province, China. A total of 71 grade-A tertiary hospitals with geriatric departments were randomly selected. Due to the resource constraints of the study, only 20% of the hospitals were targeted for sampling and the following criteria were used for sample selection: (1) promoting comprehensive medical service quality management; (2) passing ISO 9000 international quality certification for more than two consecutive times; and (3) providing long-term medical service encounters for chronic diseases in the elderly. In this study, the above three criteria were used for the sample selection, mainly because the study population must have the implementation intensity in service quality, and 15 grade-A tertiary hospitals that meet the above conditions. To achieve the purpose of this study, whose main focus was medical service encounter, this study targeted patients with chronic diseases who needed to receive hospital service encounter on a regular basis (twice a week or more) as study subjects.

First, the 15 grade-A tertiary hospitals were asked through the telephone whether they would like to participate in this study, of which 6 agreed (sample passing rate of 40%), and these 6 served as representative study cases. Second, considering the elderly with a high incidence of chronic diseases in winter and the need for high-density medical service encounters, **100 pretest questionnaires** for elderly (family members were allowed to fill out the questionnaire) experiencing long-term medical services encounter were issued from November 28–30, 2021. In total, seven invalid questionnaires were eliminated. The demographic variables of the 93 valid samples showed that most of the respondents were male patients (52.7%), with an age range of 61–70 years old (25.8%), married (72.0%), and junior college education (33.3%) as the highest education level.

The Cronbach's α of each scale is 0.81 for **Tangibles**, 0.80 for **Reliability**, 0.84 for **Responsiveness**, 0.84 for **Assurance**, 0.80 for **Empathy**, 0.93 for **Physician service encounters**, 0.89 for **Caregiver service encounters**, 0.88 for **Service personnel service encounters**, 0.90 for **Space and equipment service encounters**, 0.94 for **Patient trust**, and 0.82 for **Patient loyalty**; all of the values exceeded the recommended minimum reliability of an α of 0.7 (41–43). The alpha value was not much larger than the total value when any item was deleted, indicating that the items on each scale were homogeneous. The results indicate a high correlation in internal data sampling, and the questionnaire had high reliability for use as a formal questionnaire. Then, 700 regular questionnaires were distributed to patients and family members of six of the selected tertiary hospitals from December 2021 to January 2022. Finally, this study collected a total of 634 questionnaires. After excluding 151 invalid questionnaires (23.8%), the final number of valid questionnaires was **483**, for a valid questionnaire recovery rate of 76.2%.

## Results

### Descriptive Statistics

A total of 483 valid questionnaires were collected. In terms of gender analysis, there were 263 males and 220 females. In terms of age, the largest number of respondents were **51–60** years old (*n* = 213) and **more than 61–70 years old** (*n* = 104). Regarding marital status, the number of married subjects was 294. In terms of education level, the largest number of respondents were junior college education (*n* = 198) and junior college graduates (*n* = 147). In terms of monthly income (retirement pay), the largest number of respondents (*n* = 172) earned 4,000–6,000 RMB retirement pay. In terms of occupation (work before retirement), the largest numbers of respondents were medical industry, accounting for 24.2% (*n* = 117) of the total number of respondents, followed by those working in the service industry before retirement, accounting for about 22.4% (*n* = 108) of the total number of respondents. In terms of the number of visits/per month, the highest number of visits was 1–5 times per month, accounting for 51.6% (*n* = 249) of the total population; the rest of the respondents had more than 5 visits per month, accounting for 40.4% (*n* = 195).

### Exploratory Factor Analysis and Common Method Bias

To confirm whether the items in the initial questionnaire correspond to their potential constructs, this study used exploratory factor analysis (EFA) to assess the construct validity of the scale and examine whether an item needs to be deleted (44). We used the principal component analysis method to obtain the common interpretation variable between all the measurement questions; further, we applied the orthogonal rotation method of the equal maximum method, and took a factor load value higher than 0.4 as the basis for retaining an item. The 55 initial items in the original questionnaire were retained.

According to Qian et al. (45), the interpretation rate of the first factor should be <40%. The EFA of hospital service quality yielded a total of five factors, wherein the interpretation rate of the first factor was 31.05%. These five factors explain 74.10% of the amount of change, and the Kaiser-Meyer-Olkin (KMO) value was 0.98. The EFA of service post-encounter satisfaction yielded four factors, wherein the interpretation rate of the first factor was 32.62%. These four factors explain 73.73% of the amount of change, and the KMO value was 0.98. The EFA of patient trust and loyalty yielded a total of two factors, wherein the interpretation rate of the first factor was 38.18%. These two factors explain 71.43% of the amount of change, and the KMO value was 0.96. The above EFA results indicate that this study's sample were sufficiently internal to reasonably conduct EFA.

This study evaluated the presence of common method variance by the Harman single-factor test (46). The five factors of hospital service quality and the four factors of service post-encounter satisfaction were separately constrained to a single factor using factor analysis in SPSS. As per the unrotated factor solution, the percentage variance explained by the single factor of hospital service quality was 31.05%, service post-encounter satisfaction was 32.62%, patient trust was 34.28%, and patient loyalty was 38.13%. The above values are all lower than 50% (46).

### Reliability, Validity, and Model Fit Test

We used the reliability analysis method for corresponding measurement and test, in order to test the reliability of the scale set in the questionnaire. The Cronbach's α coefficient shows that the reliability of all dimensions and variables exceeds 0.8, indicating good reliability of the entire scale (43). The results are shown in Table 2. Table 3 shows that the model has a good fit index. Although the indexes GFI and AGFI are close to 0.9, other indexes meet the fit index, indicating a good model fit (41–43, 47).

**Table 2:** Reliability analysis (*n* = 483).

**Dimension**	**Cronbach's alpha coefficient**
Tangibles	0.892
Reliability	0.893
Responsiveness	0.897
Assurance	0.882
Empathy	0.887
Physician **service encounters**	0.930
Caregiver **service encounters**	0.907
Service personnel **service encounters**	0.887
Space and equipment **service encounters**	0.898
Patient trust	0.943
Patient loyalty	0.844

*As can be seen from Table 3, the model fit markers are good, and all markers satisfy the fitness indicator except for the markers GFI and AGFI, which were close to 0.9, indicating that the model has a good fit (41–43, 47)*.

**Table 3:** Overall Study Model Fit Pointer Analysis (*n* = 483).

**Fit**	**Name of** **marker**	**Judgment** **value**	**This study** **model**	**Marker** **conformance**
Absolute fit	CMIN/DF	<3.000	2.152	Conforming
	GFI	>0.900	0.831	Close
	RMSEA	<0.080	0.048	Conforming
Incremental fit	AGFI	>0.900	0.810	Close
	CFI	>0.900	0.994	Conforming
	NFI	>0.900	0.989	Close
Parsimonious fit	IFI	>0.900	0.994	Conforming

### Confirmatory Factor Analysis

Referring to research suggestions (43), this study conducted a confirmatory factor analysis to manage the covariance relationship between the measurement variables and their potential variables, and to test the convergence validity and discrimination validity of the measurement model. Table 4 shows that the 55 observed variables in the formal questionnaire of this study reached a significant level (T > 1.96, *P* < 0.05), with the estimated parameter factor load higher than 0.5 (48, 49), and the square multiple correlations of each item more than 0.50 (50).

**Table 4:** Confirmatory factor analysis of the research model (*n* = 483).

**Dimension**	**Measurement items**	**SFL**	**SE**	**t**	**SMC**
Tangibles	1. The hospital has safe facilities.	0.79	0.03	20.14	0.62
	2. The hospital has a barrier-free space design.	0.82	0.03	21.41	0.67
	3. The hospital has a neat and comfortable environment.	0.81	0.03	21.11	0.66
	4. The hospital has modern medical facilities.	0.78	0.03	20.04	0.61
	5. The medical staff of the hospital are professionally dressed.	0.76	0.03	19.30	0.58
Reliability	6. The doctors and nurses at this hospital can provide skilled and professional services.	0.78	0.03	20.32	0.61
	7. Doctors at this hospital can give detailed information about the patient's condition and treatment.	0.78	0.03	20.15	0.61
	8. The hospital's medical and nursing staff can provide prompt services to patients.	0.79	0.03	20.70	0.63
	9. The medical staff at this hospital have a serious and genuine attitude toward their work service.	0.82	0.03	21.64	0.67
Responsiveness	10. The hospital's medical staff is able to deal with patients' problems in a timely and prompt manner.	0.82	0.03	21.60	0.67
	11. The hospital is able to clearly inform patients of the consultation and treatment process.	0.82	0.03	21.44	0.66
	12. The hospital's doctors and nurses do not let their busy schedules delay meeting the patient's needs.	0.84	0.03	22.31	0.70
	13. The medical staff of this hospital can provide medical advice in a timely and appropriate manner.	0.80	0.03	20.97	0.65
	14. The medical staff at this hospital are always very helpful to patients.	0.84	0.03	22.42	0.70
Assurance	15. The hospital's medical staff makes patients feel safe about their visits.	0.80	0.03	20.92	0.64
	16. The professional performance of the medical staff at this hospital makes patients feel confident.	0.84	0.03	22.80	0.71
	17. The doctors and nurses at this hospital have sufficient expertise to answer patients' questions.	0.78	0.03	20.30	0.61
	18. The medical and nursing staff at this hospital generally have a good service attitude and courtesy.	0.80	0.03	21.15	0.65
Empathy	19. The medical staff of this hospital will prioritize the best interests of the patient.	0.82	0.04	21.78	0.67
	20. The medical staff at this hospital is sensitive to the privacy of the patient's visit.	0.80	0.03	20.95	0.64
	21. The hospital's medical clinic hours are convenient for the average patient.	0.82	0.03	21.72	0.67
	22. The hospital's medical staff is able to meet the special medical needs of patients.	0.81	0.03	21.51	0.66
Physician **Service encounters**	1 I think the doctors at that hospital will address my concerns.	0.78	0.03	20.42	0.61
	2 I think the doctors at that hospital are reliable for me.	0.82	0.03	21.70	0.66
	3 I think the doctors at this hospital have a good consultation attitude.	0.81	0.03	21.49	0.66
	4 I think that the doctors at this hospital possess medical expertise.	0.83	0.03	22.29	0.69
	5 I think the doctor at that hospital would recommend appropriate medication for me.	0.83	0.03	22.10	0.68
	6 I believe that the doctors in that hospital show empathy for their patients.	0.83	0.03	22.14	0.68
	7 I think the doctors at that hospital would tell the patients the treatment plan.	0.78	0.03	20.32	0.61
Caregiver **service encounters**	8 I feel that the nurses at that hospital are reliable for me.	0.82	0.03	21.82	0.67
	9 I think the nurses at that hospital will relieve me of my worries.	0.81	0.03	21.30	0.65
	10 I believe that the nurses at this hospital show empathy for their patients.	0.82	0.03	21.90	0.68
	11 I think the nurses in this hospital have a good attitude toward patient service.	0.80	0.03	21.13	0.64
	12 I think the nurses in this hospital are professional.	0.82	0.03	21.74	0.67
Service personnel **service encounters**	13 I find that the service staff at the hospital are reliable for me.	0.80	0.03	21.06	0.64
	14 I think the service staff at that hospital can relieve me of my worries.	0.83	0.03	22.06	0.68
	15 I believe that the attitude of the service staff at this hospital is beneficial to patients.	0.83	0.03	22.24	0.69
	16 I feel that the service process in this hospital is efficient and convenient for patients.	0.81	0.03	21.24	0.65
Space and equipment **service encounters**	17 I believe that the medical office of the hospital will give consideration to the privacy of the patients.	0.79	0.03	20.43	0.62
	18 I think there are clear markers in the interior of that hospital.	0.80	0.03	21.06	0.64
	19 I find that the toilets in the hospital to be clean and hygienic.	0.80	0.03	20.91	0.64
	20 I find that the consultation rooms in the hospital to be spacious and bright.	0.83	0.03	22.04	0.68
	21 I believe that the hospital has advanced medical equipment.	0.78	0.03	20.20	0.61
Patient trust	1 I have complete faith that the doctors here will provide the best course of treatment for me.	0.76	0.03	19.52	0.57
	2 The doctor who sees me at this hospital will do his or her best to provide the medical care I need.	0.80	0.03	21.20	0.64
	3 I am not worried about putting my treatment solely in the hands of the doctors here.	0.81	0.03	21.47	0.65
	4 The doctor who treated me at this hospital was very careful and attentive.	0.84	0.03	22.71	0.70
	5 The doctors at that hospital are honest about all treatment options available to me.	0.82	0.03	21.78	0.67
	6 The doctors at that hospital will think of the best way to treat me.	0.78	0.03	20.50	0.62
	7 I believe the doctors at that hospital would never mislead me about anything.	0.77	0.03	20.02	0.60
	8 I trust that the doctors at this hospital will usually use their best medical skills and efforts to treat their patients.	0.82	0.03	22.00	0.68
	9 Overall, I can trust the doctors here completely.	0.83	0.03	22.42	0.69
Patient loyalty	1 I prefer to go to the hospital for medical services	0.74	0.03	18.57	0.55
	2 I will choose the medical services provided by this hospital in the future.	0.83	0.03	22.00	0.69
	3 I am happy to recommend the medical services provided by the hospital to friends and family.	0.84	0.03	22.43	0.71

*SFL, standard factor loading; SE, standard error; t > 1.96 and p < 0.05 were significant; SMC = multiple correlation*.

Convergent validity means that the observed variables in the same construct are highly correlated with each other; therefore, these observed variables can be used to measure the same construct (51). Table 5 shows that the average variation extracted (AVE) from each facet in this study is between 0.708 and 0.747. Therefore, the measurement model of this study has convergent validity. The composite reliability ranges from 0.886 to 0.959, which conforms to the recommended value as 0.6 (50), indicating that the internal consistency of the model is high (44).

**Table 5:** Convergent validity analysis (*n* = 483).

**Dimension**	**CR**	**AVE**
Tangibles	0.924	0.708
Reliability	0.922	0.747
Responsiveness	0.926	0.715
Assurance	0.916	0.731
Empathy	0.917	0.734
Patient trust	**0.959**	**0.723**
Physician **service encounters**	0.951	0.733
Caregiver **service encounters**	0.933	0.736
Service personnel **service encounters**	0.922	0.747
Space and equipment **service encounters**	0.927	0.718
Patient loyalty	**0.886**	**0.723**

*The bold values indicated for distinguishing the dimension*.

### Correlation Analysis

Differential validity refers to the measurement of two different constructs. If the correlation degree of the two constructs is extremely low after correlation analysis, it suggest that the two constructs have differential validity (51). In terms of the discriminant validity test, this study starts with the number that the root mean square of AVE of each facet is greater than the correlation coefficient of each facet, and accounts for more than 75% of the total number of comparisons (52). First, after the correlation analysis of this study, there is a significant correlation between the constructs of the measurement model: the test of differential validity is conducted successively. The analysis results show that all constructs meet the judgment criteria, which proves that there are relevant but not the same factors among the constructs; thus, they have differential validity (Table 5).

The degree of correlation of the constructs was examined based on the Pearson correlation coefficient. All the variables were significantly positively correlated with each other at the significance level of 0.01 and the results of the correlation analysis illustrated the existence of correlation between the five dimensions of service quality and their values were all >0.700. The AVE square root value of each construct is greater than the correlation coefficient between the constructs and the scale has good comparative validity (43). The results are shown in Table 6.

**Table 6:** Correlation analysis (*n* = 483).

	**1**	**2**	**3**	**4**	**5**	**6**	**7**	**8**	**9**	**10**	**11**
1. Tangibles	**0.841**										
2. Reliability	0.744^**^	**0.864**									
3. Responsiveness	0.811^**^	0.835^**^	**0.845**								
4. Assurance	0.789^**^	0.857^**^	0.844^**^	**0.855**							
5. Empathy	0.773^**^	0.842^**^	0.834^**^	0.849^**^	**0.857**						
6. Physician service encounters	0.767^**^	0.840^**^	0.815^**^	0.852^**^	0.852^**^	**0.866**					
7. Caregiver service encounters	0.734^**^	0.815^**^	0.785^**^	0.848^**^	0.844^**^	0.859^**^	**0.868**				
8. Service personnel service encounters	0.766^**^	0.826^**^	0.794^**^	0.834^**^	0.826^**^	0.851^**^	0.856^**^	**0.864**			
9. Space and equipment service encounters	0.767^**^	0.810^**^	0.781^**^	0.811^**^	0.822^**^	0.857^**^	0.849^**^	0.844^**^	**0.888**		
10. Patient trust	0.778^**^	0.824^**^	0.803^**^	0.848^**^	0.847^**^	0.811^**^	0.850^**^	0.889^**^	0.871^**^	**0.850**	
11. Patient loyalty	0.719^**^	0.756^**^	0.743^**^	0.778^**^	0.780^**^	0.825^**^	0.810^**^	0.811^**^	0.803^**^	0.848^**^	**0.850**

^*^ *Refers to p < 0.05;*

** refers to p < 0.01; *** refers to p < 0.001; **numbers in bold** are square roots of AVE.

### Regression Analysis of Hospital Service Quality and Service Encounters

This study used linear regression to verify whether hospital service quality has an effect on healthcare contact, for which 10 separate models were developed. In this study, subject background data (including: gender, age, education, and frequency) were set as control variables, hospital service quality variable (including: tangibles, reliability, responsiveness, assurance, and care) were set as independent variables, and post-service encounter satisfaction variables (including physician service encounter, nursing staff service encounter, service staff service encounter, and space and equipment service encounter) were set as dependent variables for linear regression analysis. The results of the analysis are shown in Table 7. Models M1, M3, M5, M7, and M9 all showed that respondent age and number of visits have partially significant effects on health service encounter, indicating that the model was influenced by some of the respondent background data. In addition, models M2, M6, and M8 show that hospital service quality tangibles, reliability, responsiveness, assurance, and empathy have significant positive effects on service encounters with physicians, providers, and space and equipment, whereas model M4 only shows that responsiveness, assurance, and empathy have significant positive effects on service encounters with nursing staff; finally, model M10 shows that hospital service quality has a significant positive effect on patient service (*p* < 0.001), with a β value of 0.919. This indicates that the higher the patient's perception of hospital service quality, the higher the patient's satisfaction with the post-encounter service experience, supporting Hypothesis H1.

**Table 7:** Regression analysis of hospital service quality and satisfaction with the experience after service encounter (*n* = 483).

**Dependent variable**	**Physician service Encounters**		**Caregiver service encounters**		**Service personnel service encounters**		**Space and equipment service encounters**		Service Encounters	
	**M1**	**M2**	**M3**	**M4**	**M5**	**M6**	**M7**	**M8**	**M9**	**M10**
**Control variables**										
Sex	−0.036	−0.0^**^52	0.011	−0.009	0.052	0.040	0.008	−0.003	−0.009	−0.001
Age	−0.114^*^	−0.037	−0.135^*^	−0.055^*^	−0.094	−0.020	−0.105^*^	−0.033	−0.118^*^	−0.045^*^
Education level	−0.017	−0.023	−0.053	−0.050^*^	0.009	−0.003	−0.028	−0.043	−0.023	−0.046^*^
Number of visits	−0.065	0.030	−0.134^**^	−0.037	−0.127^**^	−0.034	−0.110^*^	−0.018	−0.115^*^	−0.024
**Independent variable**										
Tangibles		0.076^*^		0.018		0.126^**^		0.181^***^		
Reliability		0.106^*^		0.046		0.105^*^		0.107^*^		
Responsiveness		0.194^***^		0.153^**^		0.211^***^		0.170^**^		
Assurance		0.280^***^		0.362^***^		0.253^***^		0.184^**^		
Empathy		0.305^***^		0.350^***^		0.243^***^		0.290^***^		
Quality of hospital services										0.919^***^
*R* ^2^	0.013	0.809	0.026	0.784	0.025	0.773	0.017	0.751	0.020	0.852
Adj*R*^2^	0.005	0.805	0.018	0.780	0.017	0.769	0.009	0.746	0.012	0.850
*F*	1.584	222.598^***^	3.170	191.303^***^	3.040	179.111^***^	2.058	158.455^***^	2.452	548.070^***^
Durbin-Watson		1.958		1.771		1.694		1.685		1.804

* *Refers to p < 0.05;*

** *refers to p < 0.01;*

*** *refers to p < 0.001. The bold values indicated for significant effect*.

### Regression Analysis of the Effects of Hospital Service Quality on Patient Trust and Loyalty

To verify the effect of hospital service quality on trust, this study used linear regression equations to develop six separate models. In this study, subject background data were set as the control variables, hospital service quality was set as the independent variable, and patient trust and loyalty were set as dependent variables. The results of the analysis are shown in Table 8. Model M1 shows that age and number of visits have a significant effect on patient trust; model M4 shows that the number of visits has a significant effect on patient loyalty. Models M2, M3, M5, and M6 show that hospital service quality has a significant positive effect on patient trust and loyalty, with models M3 and M6 having a beta-value of 0.881 and 0.811, respectively, and a *p-*value of < 0.001. This result indicates that the higher the quality of hospital services, the higher the patient trust and loyalty. Therefore, Hypotheses H2 and H3 of this study are supported.

**Table 8:** Hospital service quality and trust and customer loyalty regression analysis (*n* = 483).

**Dependent variable**	**Patient trust**			**Patient loyalty**		
	**M1**	**M2**	**M3**	**M4**	**M5**	**M6**
	β	β	β	β	β	β
**Control variable**						
Sex	0.014	−0.001	0.004	0.006	−0.008	−0.003
Age	−0.131^*^	−0.550^*^	0.0–61^*^	−0.079	−0.010	−0.015
Education level	0.013	0.005	−0.008	0.043	0.034	0.023
Number of visits	−0.118^*^	−0.023	−0.031	−0.126^**^	−0.041	−0.046
**Independent variable**						
Tangibles		0.135^***^			0.132^**^	
Reliability		0.093^*^			0.114^*^	
Responsiveness		0.119^*^			0.089	
Assurance		0.286^***^			0.243^***^	
Empathy		0.317^***^			0.297^***^	
Quality of hospital services			0.881^***^			0.811 ^***^
***R*** ^2^	0.027	0.798	0.791	0.023	0.676	0.671
**Adj** ***R*** ^2^	0.019	0.794	0.789	0.015	0.670	0.667
***F***	3.334	207.267^***^	361.126^***^	2.808	106.568^***^	194.378^***^
**Durbin-Watson**		1.888	1.891		1.872	1.877

^*^Refers to p < 0.05; ^**^refers to p < 0.01; ^***^refers to p < 0.001. The bold values indicated for significant effect.

### Regression Analysis of Post-encounter Hospital Service Satisfaction and Patient Trust and Loyalty

This study used linear regression equations to validate the effect of ***post-***service encounter satisfaction on patient trust and loyalty in hospitals, and four separate models were developed. In this study, respondent background data were set as the control variables, service encounter (including: physicians, nursing staff, service staff and service encounter such as space and equipment) was set as the independent variable, and patient trust and loyalty were set as the dependent variables. The results are shown in Table 9. In model M1, respondent age and number of visits had a significant effect on patient trust, while in M4, the number of visits had a significant effect on patient loyalty. The results of models M2 and M5 show that patient satisfaction after service encounter has a significant positive effect on patient trust and loyalty, of which the β-values of models M3 and M6 are 0.930 and 0.852, respectively, and *p*-values are < 0.001. This result indicates that the improvement in hospital**'**s satisfaction after service encounter can enhance patients' trust and loyalty, supporting Hypotheses H4 and H5.

**Table 9:** Regression analysis of post-service encounter satisfaction with patient trust and loyalty (*n* = 483).

**Dependent variable**	**Patient trust**			**Patient loyalty**		
	**M1**	**M2**	**M3**	**M4**	**M5**	**M6**
	β	β	β	β	β	β
**Control variables**						
Sex	0.014	0.013	0.006	0.006	−0.008	−0.002
Age	−0.131^*^	−0.023	−0.021	−0.079	0.028	0.021
Education level	0.013	0.030	0.035	0.043	0.030	0.063^*^
Number of visits	−0.118^*^	−0.021	−0.011	−0.126^**^	−0.022	−0.028
**Independent variable**						
Physician service encounters		0.397^***^			0.321^***^	
Caregiver service encounters		0.144^**^			0.182^**^	
Service personnel service encounters		0.246^***^			0.193^**^	
Space and equipment service encounters		0.188^***^			0.209^***^	
Service Encounters			0.930^***^			0.852 ^***^
***R*** ^2^	0.027	0.879	0.875	0.023	0.735	0.734
**Adjusted** ***R***^2^	0.019	0.876	0.873	0.015	0.731	0.732
***F***	3.334	428.548^***^	666.221^***^	2.808	164.405^***^	263.867^***^
**Durbin-Watson**		1.956	1.940		2.003	1.998

^*^Refers to p < 0.05; ^**^refers to p < 0.01; ^***^refers to p < 0.001. The bold values indicated for significant effect.

### Regression Analysis of Patient Trust and Loyalties

This section validates the effect of patient trust on patient loyalty, for which two models were developed. In this study, respondent background data was set as the control variable, patient trust as the independent variable, and loyalty as the dependent variable. The results are shown in Table 10. In Model 1, it was found that the number of visits to the patient had a significant effect on patient loyalty, while Model 2 shows that patient trust enhancement has a significant positive effect on loyalty. This result indicates that the quality of hospital services and service encounter can enhance patient trust as well as patient loyalty. In particular, Model 2 showed that the effect of patient trust on loyalty reached significance (*p* < 0.001) with a beta value of 0.858, supporting Hypothesis H6.

**Table 10:** Trust and customer loyalty regression analysis (*n* = 483).

**Dependent variable**	**Patient loyalty**	
	**Model 1**	**Model 2**
	β	β
**Control variable**		
Sex	0.006	−0.006
Age	−0.079	0.033
Education level	0.043	0.032
Number of visits	−0.126^**^	−0.025
**Independent variable**		
Patient trust		0.858^***^
**R** ^2^	0.023	0.739
**Adjusted R** ^2^	0.015	0.736
**F**	2.808	269.449^***^
**Durbin-Watson**		2.083

^**^Refers to p < 0.01; ^***^refers to p < 0.001. The bold values indicated for significant effect.

### Verification of Structural Equation Model

In this study, LISREL 8.80 was further used for structural equation model analysis. It is assumed that the model has a good matching degree (df = 1415,χ2 = 2995.78, χ2/df = 2.12 < 5, RMSEA = 0.048, NNFI = 0.99, CFI = 0.99) (44, 51). The hypothesis of this study is verified by the estimated value of path parameters in the hypothetical structure model. When the t value of parameters is >1.65 (*p* < 0.05, single tail), the hypothetical path is established (Table 11). There are six hypothetical paths of the structural model, and the final six are established. The above indicates that hospital service quality does have a directly related impact on post service satisfaction, patient trust and patient loyalty; satisfaction after encounter does have a direct impact on patient trust and patient loyalty; patient trust does have a directly related impact on patient loyalty.

**Table 11:** Estimated values of hypothetical path parameters of theoretical structure model (*n* = 483).

**Path**	**H**	**S**	**SE**	***t*** **-value**
Quality of hospital services **→** service encounters	H1	0.96	0.07	13.36
Quality of hospital services**→** patient trust	H2	0.93	0.05	18.37
Quality of hospital services **→** patient loyalty	H3	0.90	0.05	16.99
Service encounters **→** patient loyalty	H4	1.07	0.10	11.04
Service encounters **→** patient trust	H5	0.97	0.13	7.22
Patients trust **→** patient loyalty	H6	0.77	0.25	3.14

*H, research hypothesis; S, standard coefficient; SE, standard error*.

In addition to the analysis of verifying the research hypothesis (direct effect), this study also discusses the indirect effect and total effect between facets for confirming the existing mediating effect in the research hypothesis (51). It can be seen from Table 12 that the indirect effect of hospital service quality on patient trust is significant, which indicates the phenomenon of patients' trust in hospital service quality. It will be more logical to take “hospital's satisfaction after service encounter as an **intermediary variable**. The indirect effect of ‘hospital service quality' and ‘hospital's satisfaction after service encounter” on “patient loyalty” is significant, which means that patients are loyal to the third-class hospital. Once there is the phenomenon of “satisfaction after service encounter” and “patient trust” as the **intermediary variables**, the explanation will be more reasonable. The above indicates that “satisfaction after service encounter” and “patient” **are indeed linked and indispensable** in the relationship between service quality and patient loyalty in third-class hospitals.

**Table 12:** Analysis of indirect effect and total effect (*n* = 483).

**Dependent variable**	**Independent variable**	**Indirect effect**			**Total effect**			**Supported or not**
		**SEF**	**SE**	***t***	**SEF**	**SE**	***t***	
Service encounters	Quality of hospital services	–	–	–	0.96	0.07	13.36	–/Supported
Patient trust	Quality of hospital services	1.02	0.10	10.06	0.93	0.05	18.37	Supported/Supported
	Service encounters	–	–	–	1.07	0.10	11.04	–/Supported
Patient loyalty	Quality of hospital services	0.86	0.13	6.44	0.90	0.05	16.99	Supported/Supported
	Service encounters	0.82	0.27	3.03	0.97	0.13	7.22	Supported/Supported
	Patient trust	–	–	–	0.77	0.25	3.14	–/Supported

*SEF, standardization coefficient; SE, standard deviation; t, significant level*.

## Conclusion and Recommendations

### Conclusion

**Figure 2:**
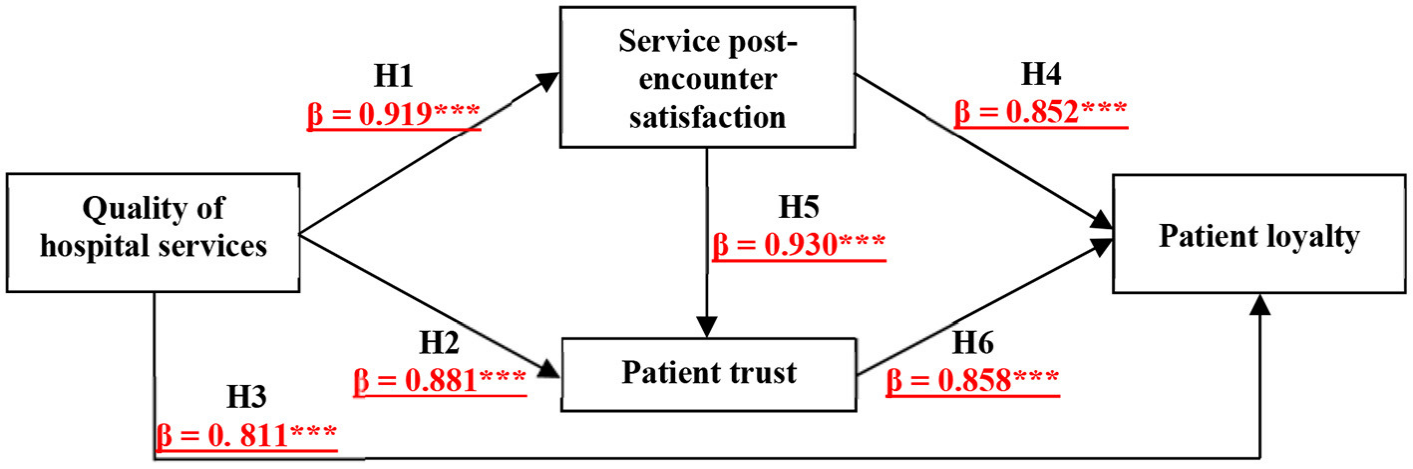
Research model analysis results. The symbol *** indicated the significant coefficients of paths.

This study was conducted with the service encounter evaluation model (14, 15) Chang et al. (21) Gonzalez (2) as a theoretical basis, and the study model and scale were further developed by combining the characteristics of healthcare service quality to explore the relationship between patients' service quality, trust, and loyalty to the hospital after patients' healthcare service encounters. The results of this study are shown in Figure 2. First, the results of the H1 analysis showed that the quality of hospital services to patients positively and significantly affects patients' perceived satisfaction after encounter with healthcare services, which means that even if the front-line doctors, nursing and service personnel try to do a good job in contacting patients, if the quality of hospital services is poor, this leads to a poor post-encounter experience of the services provided by the hospital. In other words, good service quality in hospitals also enables doctors, nurses, and service providers to create better perceptions of the service encounter among patients. In particular, the tangibles, reliability, responsiveness, assurance, and empathy of hospital service quality have a significant positive impact on service encounters with physicians, staff, space and equipment.

Second, the results of the H2 and H3 analysis revealed that the quality of services provided by frontline doctors, nurses, and service personnel in hospitals affects patients' trust and loyalty. At the same time, the results of the H4 and H5 analysis indicate that good service encounter perceptions regarding doctors, nurses, and service staff enhance patients' trust and loyalty toward doctors, nurses, and service staff. When a hospital provides high quality services, affords patients good service encounters, and gains high trust and loyalty from patients, this not only improves the quality of hospital care, but also makes patients feel satisfied and highly cooperative with the prescriptions and recommendations arranged by doctors.

In addition, a regression analysis of patient trust on loyalty showed that tangibles, reliability, responsiveness, assurance, and empathy of service quality all significantly affected patient trust. Patients were most concerned about medical equipment, the professionalism of medical staff, attitude, and consideration of patients' needs, and mistakes in these factors would reduce patients' trust, further leading to a decrease in patient loyalty. This study also found that the impact of patients' responses to service quality in terms of trust was very low and insignificant, indicating that hospital staff must improve service efficiency to hasten the resolution of patients' problems and make customers feel convenient and at ease. At the same time, the reliability of service quality in terms of patient loyalty has a very low impact and is not significant, indicating that the professional and medical skills of the medical staff need to be improved. It is recommended that the hospital conduct regular professional training and medical knowledge lectures for doctors in the hope of improving the professional skills of the medical staff and enhancing the hospital's own competitiveness.

Finally, satisfaction after the service encounter also significantly affects patient trust, so hospitals that implement good medical equipment and overall comfort, robust care, warm care, and friendly healthcare staff can significantly increase patient trust and loyalty. The results of the H6 analysis showed that the quality of services provided by the hospital significantly enhances patient trust and further increases patient loyalty to the hospital. The results of this study also revealed that the tangibles and reliability of service quality had a very low and non-significant impact on the service encounter with nursing staff, indicating that the medical equipment, environment, and medical professionalism of healthcare staff need to be improved when encountering patients. Additionally, a comparison of the strengths of the service encounter perspective between this study and previous studies is described clearly in Table 13 to better understand the research novelty.

**Table 13:** The comparison of the strengths of this study with previous studies using the service encounter perspective.

**Source**	**The previous studies**
Fatima et al. (13); Rostami et al. (12)	Focused on an assessment of patient loyalty using service quality.
Al-Neyadi et al. (14); Behdioglu et al. (15); Owusu Kwateng et al. (11)	Focused on an assessment of patient satisfaction by engaging service quality.
Alhatti (16); Fatima et al. (13); Miao et al. (17); Rostami et al. (12)	Confirmed patient satisfaction and patient loyalty as a positive relationship
Fatonah (18); Hajikhani et al. (19); Huang et al. (20)	Established the relationship between physician-patient and patient loyalty
Alhatti (16); Fatima et al. (13)	Established the relationship between the quality of the patient-patient and patient loyalty
**Source**	**Strengths of the present study**
Present study	Proposed a comprehensive research model based on the “Service Encounter Assessment Model,” and integrating tertiary hospital characteristics. Provided an evidence-based practice study using a service encounter perspective for representative case tertiary hospitals in China. Remedied research gaps of previous studies, the “service encounter” perspective in healthcare is utilized to explore the relationship between hospital services quality, patient trust, and patient loyalty. We studied elderly patients, who experience long-term medical service encounters, generate experience feelings, which positively influence patient trust and loyalty.

### Managerial Implications

The five dimensions of hospital service quality can positively and directly affect the performance of post-service encounter satisfaction almost across the board, except for tangibles and reliability, which have no significant effect on post-service encounter satisfaction of nursing staff. This may be because caregivers are predominantly presented as assisting physicians from the sidelines, causing patients to dilute their perceptions of these two dimensions of the caregiver service in scenarios where physicians and caregivers are both present. For hospital management, the focus could be on the direct assistance provided by the nursing staff to the patient, in which nursing staff not only share the work of the physician, but also better complement nursing and medical care.

The five dimensions of hospital service quality also positively and directly impact patient trust and loyalty almost across the board, with the exception of the responsiveness dimension, which has no significant impact on patient loyalty. This may be because patient loyalty is more focused on the ability to resolve a condition carefully than on the expectation that the physician will complete the consultation in a short period of time. For hospital management, consideration should be given to encouraging physicians to extend consultation durations and limiting the number of patients registered by some physicians.

All four dimensions of post-encounter satisfaction positively and directly impact patient trust and loyalty, and all have the greatest impact on post-encounter satisfaction with physician services. Hospital administrators should continue to maintain the quality of not only the physician talent they employ, but also the quality of the nursing staff, service staff, space and equipment, and the maintenance of updated hardware.

Finally, in an era of increasing competition in the medical services industry, patients retain a preference for tertiary hospitals because of the greater injection of public resources into the medical standards of their professionals and facilities, which boosts patients' confidence that they will receive good medical treatment. Therefore, the accreditation system of tertiary hospitals will enable them to maintain their designation so that they can sustainably provide the appropriate level of care and contribute to society.

In China, regardless of being laypersons or medical workers, when it comes to hospitals, everyone agrees that grade-A tertiary hospitals are the best. These hospitals are the first choice to treat chronic diseases and physical discomfort. Grade-A tertiary hospitals are also the first choice for the employment of doctors and nurses. Given the continuous improvement of patient awareness, medical demand is more significant than medical supply. In addition, the frequent occurrence of various large-scale environmental, infectious diseases, and chronic diseases in recent years, the medical management system of grade-A tertiary hospitals should take the most streamlined human resources for the quality and quantity of medical services and adopt a “patient-oriented” business philosophy. In this study, we suggest that hospitals create “patient satisfaction” and “service encounters” for improving “patient trust” and “patient loyalty” to realize the ultimate ideal of sustainable operations of medical institutions.

### Theoretical Implications

Population aging is a social problem faced by many countries, including China. The consequent pressure on the medical system comes from the increase in elderly patients with chronic diseases. Elderly patients with chronic conditions are bound to seek medical resources periodically. In China, grade-A tertiary hospitals are the best places to provide medical treatment for such people. Therefore, medical service contact has become an essential issue between grade-A tertiary hospitals and elderly patients with chronic diseases. Elderly patients with chronic diseases traditionally prefer physical medical experiences and are more sensitive to people and things in biological and medical treatment than young people. This study employed post-service encounter satisfaction and trust as mediating variables and explained the effector pathways of hospital service quality with fairly positive and significant results. Hospital service quality is intangible, manifested through tangible hospital personnel (doctors, nursing staff, service staff), as well as space and equipment, and perceptions of patients, which in turn leads to patients' trust in hospital care and ultimately to loyal attitudes.

This result adds to the service quality theory. Discussions on service quality in the past frequently refer to the impact of the perceived tangibles, reliability, responsiveness, assurance and empathy of the service provider on subsequent constructs, such as trust and loyalty, and few explore the actual people, events and variables that the service recipient encounters, such as medical staff, service personnel, and space and equipment. The mediating role of post-service encounter satisfaction in this study specifically indicates that regardless of the degree of medical service quality provided by medical institutions, hospital service quality can affect loyalty not only directly and positively (direct effect, 0.811) and through patient trust (indirect effect, 0.881 × 0.858 = 0.756), but also from post-service encounter satisfaction (indirect effect, 0.783, 0.919 × 0.852 = 0.783), and service post-encounter satisfaction triggering patient trust and loyalty (indirect effect: 0.733, 0.919 × 0.930 × 0.858 = 0.733), thus providing a complete explanation of the mechanisms by which hospital service quality will result in patient loyalty. Therefore, grade-A tertiary hospitals will strive toward achieving customer orientation.

### Study Limitations and Suggestions for Future Studies

From the results of the overall model analysis of this study, we found that patients showed a positive relationship between hospital service quality, post-service encounter satisfaction, trust, and loyalty for the six tertiary hospitals selected for the study. This study also confirmed that hospital service quality and the post-service encounter satisfaction of healthcare personnel play a decisive and important role in the improvement of patients' trust and loyalty. However, this study has some limitations, such as in the selection of scales. For example, there may be a potential effect of nursing staff on patient trust and loyalty under the influence of service encounter and the factor of patient-medical staff relationship commitment. In addition, there could be a possible mediating effect on patient-medical staff relationship commitment between the quality of healthcare services and patient satisfaction. Therefore, it is suggested that future studies explore the patient-medical staff relationship in greater detail. In summary, this study suggests that hospitals must focus on enhancing service quality, as good service quality is more effective in ensuring patient satisfaction with the service encounter and increasing patient trust. When patients are highly cooperative with doctors in arranging prescriptions and advice, the higher the loyalty to doctors, nurses, and service providers, which means that patients feel convenient and reassured, and strengthen their dependence (5, 15). With such a positive feedback loop, it is believed that the recovery rate of the patients will be improved.

## Data Availability Statement

The raw data supporting the conclusions of this article will be made available by the authors, without undue reservation.

## Ethics Statement

Ethical review and approval was not required for the study on human participants in accordance with the local legislation and institutional requirements. Written informed consent for participation was not required for this study in accordance with the national legislation and the institutional requirements.

## Author Contributions

A-JS: writing—original draft and revising, research conceptualization, methodology, and data analysis. Y-FH: research conceptualization, methodology, supervision, coordinating tasks, and writing—revising the manuscript. G-YL and MY: formal analysis and validation, investigation, writing—revising the manuscript, and final approval of the version. W-YL, Y-YD, and Z-HS: research administration for the empirical project, resources, investigation, interpretation of data, writing—revising the manuscript, and final approval of the version. YW: communication with research objects, data collection, writing—revising the manuscript, and final approval of the version. All authors read and approved the final manuscript and agreed to be accountable for all aspects of the work in ensuring that questions related to the accuracy or integrity of any part of the work are appropriately investigated and resolved.

## Funding

This research was supported by the starting research fund for higher-level talents from Huaqiao University [Grant Number: 21SKBS007], the Social Science Fund of Jiangsu Province [Grant Number: 19SHB003].

## Conflict of Interest

G-YL was employed by Shandong Holyscape Marketing Research & Consulting Co., Ltd. The remaining authors declare that the research was conducted in the absence of any commercial or financial relationships that could be construed as a potential conflict of interest.

## Publisher's Note

All claims expressed in this article are solely those of the authors and do not necessarily represent those of their affiliated organizations, or those of the publisher, the editors and the reviewers. Any product that may be evaluated in this article, or claim that may be made by its manufacturer, is not guaranteed or endorsed by the publisher.
